# Sanitation Improves Stored Product Insect Pest Management

**DOI:** 10.3390/insects10030077

**Published:** 2019-03-17

**Authors:** William R. Morrison, Alexander Bruce, Rachel V. Wilkins, Chloe E. Albin, Frank H. Arthur

**Affiliations:** 1 USDA, Agricultural Research Service, Center for Grain and Animal Health Research, 1515 College Ave., Manhattan, KS 66502, USA; alexander.bruce@ars.usda.gov (A.B.); frank.arthur@ars.usda.gov (F.H.A.); 2 Department of Entomology, Kansas State University, 123 Waters Hall, Manhattan, KS 66506, USA; rvw@ksu.edu; 3 Department of Engineering, Kansas State University, 1046 Rathbone Hall, Manhattan, KS 66506, USA; albinc@ksu.edu

**Keywords:** sanitation, integrated pest management, behavior, efficacy, chemical control, biological control, cultural control, exclusion, monitoring

## Abstract

There is a large suite of insects that attack anthropogenic agricultural goods after harvest. Proper sanitation programs for food facilities are now recognized as the foundation of good integrated pest management (IPM) programs for stored products throughout the post-harvest supply chain. While good sanitation programs are generally thought to reduce the abundance and diversity of insects, there has been less appreciation of the manifold ways that sanitation interacts with a range of other IPM tactics to modulate their efficacy. Here, we review the literature on how the effectiveness of chemical, physical/cultural, biological, and behaviorally-based control tactics varies with changes in sanitation. In addition, we discuss how sanitation may affect ongoing pheromone- and kairomone-based monitoring programs. Where possible, we quantitatively compile and analyze the impact of sanitation on the fold-change in the efficacy of IPM tactics. We found that decreased sanitation negatively affected the efficacy of most tactics examined, with a mean 1.3–17-fold decrease in efficacy under poorer sanitation compared to better sanitation. Sanitation had neutral or mixed impacts on a few tactics as well. Overall, the literature suggests that sanitation should be of the utmost importance for food facility managers concerned about the efficacy of a wide range of management tactics.

## 1. Sanitation Historically and Its Contemporary Significance

Long before the beginning of human agriculture about 10,000 years ago, there was a large suite of insects that attacked trees, logs, dead or rotting fruit, and caches of resources created by other species [1]. As humans began producing surplus agricultural commodities, there was the need to store these safely and for longer time periods, which first led to the rise of granaries in agricultural areas [2], then later to the proliferation of a wide assortment of anthropogenic structures to store, transport, process, and market post-harvest products. During this process, many insects continued feeding on their preferred natural hosts but switched locations to these anthropogenic structures where their food was much more abundant than in natural landscapes [3]. It is estimated that between 8–70% of the world’s food is lost to insects and other pests after harvest, depending on location and resources for post-harvest pest management [4]. In 1991, the economic losses to post-harvest products from insects alone were estimated to be USD $5 billion [5], which would be almost USD $9 billion in today’s money given the U.S. Bureau of Labor Statistics’ average inflation rate of 2.28% per year. Globally, post-harvest losses account for over $100 billion [6].

**Figure 1:**
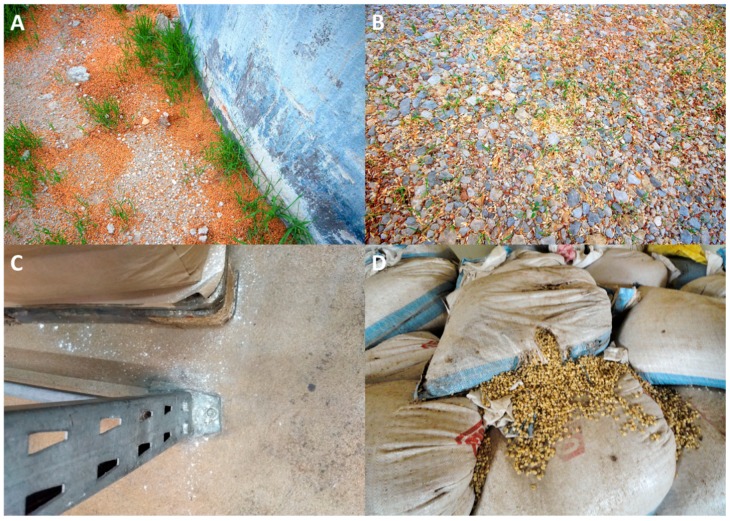
Common sites of spillage in and around food facilities, particularly (**A**) directly adjacent to a grain elevator in the US, (**B**) outside away from buildings in the US, (**C**) inside an industrial bakery in Germany next to packaged flour, and (**D**) inside a warehouse in Ghana from compromised packaging. All of these are ideal habitats for reproduction of insects. Photo credits: Drs. Rob Morrison and Frank H. Arthur, USDA-ARS.

As commodities proceed through the post-harvest supply chain, small or large grain spills may occur, food dust may accumulate, and residual rubbish heaps may be located near facilities that amass more host materials and refugia (Figure 1) [7]. For example, during the warm season, densities of rice weevil (*Sitophilus oryzae* (L.); Coleoptera: Curculionidae), rusty grain beetle (*Cryptolestes ferrugineus* (Stephens); Coleoptera: Laemophloeidae), and saw-toothed grain beetle (*Oryzaephilus surinamensis* (L.); Coleoptera: Silvanidae), quickly increased in the boots, pits, and load-out areas of grain elevators and feed mills [8]. A partial budget analysis revealed that cleaning the elevator boot every 30 days resulted in 5.2 times more revenue than the costs of sanitation for a 6.4 metric ton load of wheat, derived in part by avoiding grain discounts from decreased grain quality, presence of live insects, and rejected loads [9]. In addition, as grain and other commodities are emptied from bins and warehouses, they may be incompletely cleaned, leaving behind residual commodities. This may also be true of debris surrounding food facilities. For instance, in Europe, 60–75% of debris around food facilities in the former Yugoslavia were infested with stored product mites [10]. In nine commercial elevators in Kansas, pest insects were observed in 41% of over 1500 samples taken, and 42% of these samples were less than 1.5 kg, with more insects per kg of residue observed in smaller patches than larger ones [11]. All these factors (spillage, residues, trash heaps, incomplete cleaning) may act as source populations for future infestations of commodities.

Pest insect movement may spread insect infestation when sanitation is poor in and around that facility. Many stored product insects, including *Rhyzopertha dominica* (F.) (Coleoptera: Bostrichidae), are known to have natural refugia in the landscape beyond food facilities, as they were originally wood borers on trees [12]. This and other species will readily disperse large distances in order to seek out and immigrate into food facilities [13], and they may easily colonize points of spillage as intermediary stops along the way. Prior work has also recaptured *Trogoderma variabile* Ballion (Coleoptera: Dermestidae) and *Plodia interpunctella* (Hübner) (Lepidoptera: Pyralidae) at distances of 75 m and 136 m away from the points at which they were marked, and found that *T. variabile* is able to immigrate into food processing plants from the surrounding environment [14]. Another study found that these same two species were consistently captured outside of flour mills, and that captures were not affected by fumigations, suggesting that these species may be dispersing from the landscape [15]. A variety of stored product species are regularly captured outside of food facilities [16,17], and research suggests that effective exclusion, such as the use of gaskets, can contribute the suppression of immigration of insects into these structures [18]. More recently, long-lasting insecticide netting has shown promise as another exclusion method, as it is able to significantly reduce movement and impair the dispersal capacity of adult stored product insects after brief contact with the netting [19]. These and other studies suggest that the degree of sanitation and the movement of insects may interact to worsen or improve pest suppression in food facilities.

The effects of poor sanitation may be magnified as commodities proceed through the post-harvest supply chain, resulting in greater infestations as commodities near the end of the chain and are bought by consumers. For instance, in eight commercial Kansas pet food stores, researchers found 19 species of stored product insects belonging to 20 families through pheromone- and food-baited traps [20]. The same study found an average of 65–650 adult insects/kg of bagged pet food products on store shelves. In sampling over five years in Greece, almost 80% of samples were infested with stored product insects in commercial stores, which had the highest proportion of infested samples compared to other types of food facilities (cooperative unions, farm stores, flour mills, and silos) for animal fodder, flour and bran, edible products, residues, and other various products [10].

The biology of specific species may interact in other ways with sanitation that are often deleterious for pest management. For example, *Trogoderma glabrum* (Herbst) (Coleoptera: Dermestidae) typically undergo 5–6 larval instars when food is abundant, but when they are deprived of food for extended periods, they began to molt in reverse, a process known as retromolting [21]. During a full year of food deprivation, larval *T. glabrum* underwent 5–8 retromolts, but underwent these irregularly. To compensate for water loss from molting, the larvae are able to absorb atmospheric humidity. If larvae contact food spillage or small amounts of residue, they can proceed to molt progressively, but will begin to retromolt if food becomes limiting. They can cycle back and forth between progressively and retrogressively molting for over two years [21,22]. Other species of stored product insects may make their own metabolic water while feeding and have extreme adaptations to conserve water loss [23]. For these insects, including *Callosobruchus* spp. [23], feeding on spillage sites in locations with potentially more moisture may increase the fitness of individuals that are already adapted to living in an otherwise highly xeric environment. Moreover, some species seek out the edges of enclosed areas, such as *Tribolium castaneum* (Herbst) (Coleoptera: Tenebrionidae) [24], and because food dust and spillage may accumulate in these areas, there is an increased risk of infestation when sanitation is not a priority. Under conditions where a source of food or spillage is infested with a high density of *T. castaneum*, cannibalism may become more frequent [25], and conspecifics may be induced to disperse elsewhere [26], resulting in spreading infestations in a facility. There are a variety of other adaptations in physiology, development, and life history, which may benefit stored product insects in environments where sanitation is ignored or deemphasized.

In the modern era, sanitation is now recognized as the foundation for many integrated pest management (IPM) programs for stored products by industry personnel (e.g., [27,28,29]). Common IPM tactics to improve sanitation in food facilities include increased stock rotation, using crack and crevice treatments with residual chemical products, regularly sweeping, vacuuming and removing food dust and debris, closing doors and windows, use of air curtains near entrances, screening, and space treatments that include fogging with aerosols. However, there is less appreciation for how sanitation affects not only overall pest abundance but interacts with a variety of other pest management and monitoring techniques, either by decreasing or increasing their efficacy depending on the sanitation level in the anthropogenic environment. The goal of this review is to specifically understand how and to what extent sanitation may hinder chemical, physical/cultural, and biological control, as well as behaviorally-based management strategies and pest monitoring in the post-harvest supply chain. In particular, we review how sanitation affects a large variety of post-harvest pest management techniques, including fumigation, aerosols, grain protectants, aeration, grain chilling, heat, modified atmospheres, biological control, and pheromone-based techniques.

## 2. Materials and Methods

We have reviewed the primary scientific literature using Google Scholar and Web of Science. We used key search words, including but not limited to “sanitation,” “presence of food”, “chemical control”, “physical control”, “biological control”, “pest management,” “effectiveness,” “monitoring,” “pheromone-based,” “sanitary,” and “trapping” in various combinations. All relevant literature was compiled into a database and summarized in Table S1. In total, we reviewed 117 peer-reviewed, proceedings, and bulletin articles on sanitation, and summarize the main results below. We classified each pest management tactic under one unique category of IPM tactics, which included chemical control, physical/cultural control, biological control, monitoring, or behaviorally-based management. For quantitative data, we pulled information for each experiment in relevant studies (*n* = 1647 independent observations from experiments in 48 studies), and calculated the fold-change in efficacy of the tactic in direct comparisons between when food was present or absent, or in cases where there was less or more food. To give an illustration of this calculation, if survival of a species was found to be 10% for an insecticide in the absence of food compared with 90% when food was provided, this would be a net negative 9-fold change in efficacy (e.g., 90/10 = 9) of the tactic. Similarly, if average mortality from a modified atmosphere is 80% with only 1 g of food provided, but decreases to 40% when 10 g of food is provided, this would be a net negative 2-fold change in efficacy (80/40 = 2) of the tactic. These are the numbers that appear in Table 1. For studies where data was presented only graphically, we used ImageJ to retrieve the appropriate values [30]. We separated results by life stages (as possible), and recorded the location information of data collection, target species for management, and the tactic employed. These were used as explanatory variables in an ANOVA to determine the fold-change in control efficacy under poorer sanitation. We excluded levels of a factor if there were less than 3 independent observations from the literature. Upon a significant result from the model, Tukey HSD was used for multiple comparisons. All tests were performed in R Statistical Software [31] with α = 0.05. Not all the literature we review is included in the quantitative analysis portion of this study, because (1) presence of food on tactic efficacy was not directly evaluated, or (2) such a test is not possible. For some situations, such as grain chilling and aeration, tactic efficacy on bins with commodity was compared to control bins lacking the tactic but still containing the commodity, which would represent what would happen to a space with an abundant food source (e.g., poor sanitation) in the absence of the tactic. It is not possible to include every reference in this review, thus any mention of a publication is largely for the purposes of illustrating concepts and ideas.

## 3. Sanitation and Chemical Control

The effect of sanitation on the efficacy of control varied by tactic (*F* = 29.6; df = 9, 1170; *p* < 0.0001), but had an almost uniformly negative effect where data is available (Table 1). The majority of results from the studies reviewed here (e.g., a total of 1240 out of 1647) have focused on the effect that presence of food has on chemical control methods. For most kinds of chemical control, decreased sanitation (defined as food present or more abundant) had a pronounced negative direct effect on the efficacy of the management tactic (Table 1). Overall, decreased sanitation reduced chemical control tactics’ efficacy by an average of 8.5-fold. Even in the case (e.g., grain protectants) where sanitation had less influence, sanitation may hamper the tactic’s overall usefulness through indirect effects. The main areas of chemical control where literature was available on effects of sanitation were fumigants (methyl bromide, phosphine, sulfuryl fluoride, etc.), aerosols (liquid insecticide formulations mechanically atomized and dispensed at small particle size, including fogging and misting), residual treatments (crack and crevice, pre-binning treatments, other surface sprays, insecticidal powders and dusts), grain protectants (sprayed directly onto grain directly, usually as it is loaded into bins), and modified atmospheres (N_2_, ozone, CO_2_, low O_2_). Below, we review the main findings for each one of these methods in turn.

Decreased sanitation reduced fumigant efficacy by almost 16-fold (Table 1). When assessing the efficacy of a fumigant, especially for structural applications, it is important to understand a fumigant’s effectiveness at inducing mortality through a vertical column of commodity. This is because there may be deep accumulations of a commodity that cannot be penetrated by the fumigant during structural fumigation, for example from accumulated residue in wall voids, in or on equipment, in deep cracks in the foundation, and in other locations. Some of these large columns of commodity may provide effective refugia for insects to escape fumigation, and then reinfest a structure. For example, when ethyl formate was combined with carbon dioxide in a 50 kg column of wheat, it killed *T. castaneum* and *S. oryzae* equally well regardless of whether they were located at the top, middle, or bottom, of the column of wheat [32]. Leesch [33] found that phosphine could penetrate a 4.12-m column of wheat, but did so faster and at greater concentration with carbon dioxide, suggesting potential high efficacy for structural applications to overcome poor sanitation. Because of their intrinsic gaseous properties, fumigants can penetrate most commodities, even those as dense as wood [34]. Even so, there can be difficulties with achieving the appropriate exposure time and/or an inability to reach a lethal gas concentration for a sufficiently long period at required depths. This has special relevance for structural applications of fumigation, especially in cases where sanitation is an issue. Moreover, different fumigants vary in their ability to penetrate dense materials. The main risk of poor sanitation to the use of fumigants may be a combination of risk of surviving insects in the fumigated space and/or the risk of recolonization from adjacent infested commodities or areas. These dual risks should not be understated, as prior work has found that after fumigation, stored product insects (including *T. castaneum*) will regularly and repeatedly increase in population until the next fumigation [15]. Other work has found that regardless of fumigant compound or time of year, the facilities that maintain the highest sanitation levels, also achieved the longest rebound time, thus maximizing tactic efficacy [35]. Thus, poor sanitation will likely increase the rate of insect increase, decrease the time between fumigations, and cost food facilities additional money in more frequent shutdowns for fumigation.

Grain protectants seem to be the least directly affected by sanitation (Table 1). For example, there was no progeny production for insecticide-exposed *Tribolium confusum* du Val (Coleoptera: Tenebrionidae) adults when placed on wheat treated with beta-cyfluthrin, alpha-cypermethrin or deltamethrin [36]. However, when insecticide-exposed *T. confusum* adults were subsequently exposed to untreated flour for a 7-d interval, progeny were produced, but only for cypermethrin-exposed adults only. A specific grain protectant may be more or less effective on a certain commodity. Kavallieratos et al. [37] found that there were 4–22, 2–16, and 2–15 times more *S. oryzae*, *R. dominica*, and *T. confusum*, respectively, on rice treated with 1 ppm or less of chlorfenapyr after 60 d than barley, maize, or wheat. As grain protectants are designed to protect bulk grain and be applied directly to a food source, they are less susceptible to failures from sanitation. For example, Collins et al. [38] found that field-collected populations of *Cryptolestes* spp., *S. oryzae*, and *T. confusum* contained no surviving insects after exposure to a discriminating dose of fenitrothion applied to wheat. The main threat to the efficacy of grain protectants may be from poor sanitation of bins after off-loading grain (leaving untreated grain refugia in a bin or silo), or through increased opportunities for infestation of a bin over a long storage period from poor sanitation around a facility after the residual activity of the grain protectant attenuates.

Aerosol treatments were consistently negatively affected by poor sanitation (Table 1), with an almost 3-fold reduction in efficacy under poorer sanitation regimes compared to better ones. Arthur and Campbell [39] found that the survival of *T. confusum* after an aerosol application (pyrethrins and piperonyl butoxide) in a warehouse increased linearly with the amount of flour present over 0–2000 mg. On average, the presence of the maximum amount of food in that study increased survival of adults by 3–8-fold after 2–14 d compared to controls without food. Likewise, Toews et al. [40] also found that the mortality of *T. castaneum* increased linearly with increasing amounts of flour, and found that mortality after exposure to aerosolized esfenvalerate or pyrethrins never exceeded 60% in dishes containing petri dishes with 2 g of flour. This represented a greater than 2-fold decrease in efficacy. Similarly, the percent of active *T. confusum* 14-d after exposure to a pyrethrin aerosol was 2- to 4-fold greater when they were given access to food afterwards than when food was absent [41]. Indeed, Arthur et al. [42] assessed the ability of regular 2-wk and 4-wk esfenvalerate aerosol applications to control an infestation of *T. castaneum* from flour food patches underneath a series of shelves in a simulated warehouse, and found that even over 20 weeks, populations of all combined life stages were equivalent to warehouses without aerosol applications. This highlights the fact that the absence of appropriate sanitation measures may contribute to aerosol treatment failures.

Likewise, residual insecticide treatments were negatively affected by poor sanitation. In total, there was an almost 11-fold decrease in efficacy under poorer sanitation regimes for this tactic (Table 1). For example, one study examined a wettable cyfluthrin powder and found that the percent of a concrete arena covered with flour or sawdust prior to exposure of *T. castaneum* significantly affected both knockdown and survival at subsequent time intervals [43]. Only 20–40% of the arena needed to be covered to result in a significant increase in recovery and survival of beetles, reducing the efficacy by 2-fold to 10-fold. Another study examined the efficacy of chlorfenapyr after exposing *T. castaneum* for 2 h, and found that the presence of food increased survival by 3–10-fold at the end of 7 d [44]. In bulk grain, Athanassiou et al. [45] found that a surface layer treatment of methoprene was not as effective at decreasing *R. dominica* progeny as a whole-grain treatment for wheat and rice. In thinking about how this may apply to structural treatments, if residual insecticides are used and there are refugia available (e.g., below the top layer of commodity in areas of residue accumulation), this suggests that the effectiveness of chemical control tactics against stored product insects may be decreased. Salama and Abdel-Razek [46] found that the amount of δ-endotoxin needed to control *P. interpunctella* doubled when the toxin was mixed with whole wheat or corn compared with a control diet. However, sometimes the effects can be variable, as a different study found that mortality was not affected by the presence of food for any of the seven species of stored product insects exposed to beta-cyfluthrin and imidacloprid or beta-cyfluthrin alone [47]. Nonetheless, most studies document a pronounced negative effect of poor sanitation on the efficacy of residual treatments in controlling stored product insects.

Modified atmospheres have been specially designed for penetration of commodities to kill insects on or in food refugia, which is supported by the low change in direct efficacy under poorer sanitation regimes found in this review (Table 1), but they also encounter their own intrinsic indirect limitations under poor sanitation regimes. Most work with modified atmospheres has been evaluated in the context of bulk grain storage. Different modified atmospheres may penetrate different commodities at different rates and be more or less successful at inducing mortality of post-harvest insects. For example, ozone is very reactive, which restricts its movement into a grain mass via a process known as the ozone demand of the medium [48]. This may partly explain why the mortality of *Ephestia kuehniella* eggs decreased by almost 20-fold when placed at the bottom of a 2 kg lot of grain compared to the top of the grain mass [49]. The same study found that eggs, pupae, and adults were tolerant to ozone application at the bottom of the grain mass, and survival was high. This sometimes limited ability to penetrate a commodity may be most important in structural applications of modified atmospheres, especially in structurally complex environments under poor sanitation regimes, which may present large amounts of residue that cannot be effectively penetrated by the compound. Likewise, Athanassiou et al. [50] found that ozone was less effective when insects were in grain masses compared with empty spaces. At a depth of 2.7 m into a 0.75 metric ton grain mass, there was a 15% reduction in the amount of ozone compared to where the ozone was introduced in the headspace [51]. This suggests that for structural applications, ozone may have difficulty penetrating wall voids and other harborages of insects. More generally, these studies illustrate how analogous dynamics may be important when considering the effect of sanitation on structural treatments of facilities. Even if modified atmospheres are not commonly used for structural treatments presently, one can imagine that if ozone (or any fumigant) has difficulty penetrating areas where food residue has built up, it may leave refugia for stored product insects to survive, which may then be able to reinfest a facility. This emphasizes the potential importance of sanitation for the application of modified atmospheres for pest management in post-harvest systems.

Other modified atmospheres have similar limitations, even if they are less reactive with the exposed substrates. For example, when N_2_ was added at the bottom of a 100 cm vertical grain column composed of wheat, an O_2_ gradient between 0.9% at the bottom and 18.5% near the top was formed [52], with no apparent effect on the movement on a variety of species of post-harvest insects. Prior work has shown that O_2_ concentrations need to be decreased to between 1–3% to induce mortality in stored product insects [53]. This suggests that for structural applications of N_2_, insects may be able to find refugia in deep accumulations of food residue under poor sanitation regimes. One major limit to the use of modified atmospheres is that the area being treated must be relatively airtight, usually determined by negative pressure testing [54]. The use of CO_2_ fumigation to control stored product insects has been relatively successful for large masses of grain (21.5–209 MT of wheat: [55,56]), but the key is that these structures need to be fully sealable. Thus, sealing structures or a space may ameliorate the limitations of modified atmospheres under poor sanitation regimes.

There are a variety of possible explanations for why decreased sanitation may lead to worsened outcomes for pest management for chemical control. First, the food substrate may affect the chemical control measure directly by forming a barrier so that insects do not encounter the residues. For contact residual insecticides, this may be a key process (e.g., [43]). For chemical control measures that can diffuse through air such as fumigants, this factor may be less important, though may still play a role if penetration is an issue, as may be the case for especially dense commodities or when the gas is especially reactive with the commodity (e.g., ozone; reviewed in [54]). Relatedly, clean food may provide an abrasive surface, which can act to scrub insecticide particles off an insect’s cuticle after it is exposed [43]. This latter possibility is supported by a study that found there was a 4- to 5-fold greater number of *T. castaneum* and *O. surinamensis* killed in whole wheat treated with spinosad compared to either cracked wheat or wheat flour treated with the same concentrations of compound [57]. These mechanisms still need to be directly tested to understand their relative importance. However, together, these physical protection hypotheses may help to partially explain the benefits of sanitation in specific circumstances. There is also a potential physiological protection hypothesis to explain the benefits of improved sanitation for chemical control measures. Namely, as insects are provided with an additional source of nutrition, it enhances the ability of the insect to detoxify the insecticide metabolically, or may more generally upregulate expression of all or a select group of genes that increase the likelihood that the insect will recover after exposure. Insect feeding may result in changes to a large number of genes in some insects (e.g., [58]), though to our knowledge, this has never been evaluated specifically in stored product insects. While all of these hypotheses may be acting concurrently, no study has rigorously tested them in stored product insects to our knowledge. Regardless of the mechanism, it is clear that sanitation plays a key role in improving the effectiveness of many post-harvest chemical control tactics.

## 4. Sanitation and Physical/Cultural Control Measures

While there have not been as many studies investigating the effect of sanitation on physical and cultural control measures (294 out of 1647 results), there are still important cascading effects from changes in sanitation on these tactics. There are a variety of techniques considered physical or cultural control techniques, including temperature modification (heat, grain chilling, or aeration treatments), passive methods (packaging, screening, hermetic storage, air doors, etc.), active methods (inert dusts, sieving, other mechanical disturbance, etc.), and electromagnetic methods (ionizing radiation, microwaves, and radio frequencies) (e.g., reviewed in [59]).

Temperature modifications may be somewhat buffered from slight changes in sanitation, but very poor sanitation and build-up of food debris may readily lead to inadequate control of insects for heat treatments, with an average 14-fold decrease in tactic efficacy. For example, Subramanyam et al. [60] demonstrated that areas with flour deeper than 2 cm have close to 0% adult mortality of *T. castaneum* from heat treatments with forced air gas heaters in a flour mill compared to almost 100% mortality at the surface of the flour. By contrast, for the same depth comparison, there was a 20% decrease in egg mortality compared to the surface. Another comparison of *T. castaneum* life stages generally found that by a flour depth of 1 cm, efficacy of heat treatment had decreased by 10% for eggs and 63% for adults [61]. This suggests that flour is a poor conductor of heat. Other commodities may differ in their capacity to conduct or allow passage of heat, with larger particles likely allowing more heat to diffuse. However, other research has shown that even whole wheat kernels may have an insulating effect during heat treatments [62], thus emphasizing the importance of good sanitation practices.

Conversely, for extreme low temperature, the goal of grain chilling and aeration methods has not been to induce mortality of post-harvest insects, but rather to cool a grain mass to levels that would inhibit insect development [63,64,65]. Grain chilling and aeration have most commonly been considered for bulk grain storage. On average, in comparing grain masses with insects and no cooling to grain masses with insects and the presence of cooling mechanisms, there was an almost 32-fold increase in efficacy of control for stored product insects (Table 1). Though infrequently used for structural treatments, it is clear from this data that if there are residues in bins, or areas of spillage in a facility, cooling these areas may dramatically reduce the speed of development of stored product insects. While grain chilling can be cost prohibitive, it is highly effective in dramatically slowing the development of insects in the affected area, even in bulk grain storage. Grain aeration has a better cost-benefit ratio [66], and may also be effective at slowing the development of insects [67], especially when the air temperature outside the food facility or silo is lower than within. For example, Flinn et al. [68] found that automatic grain aeration begun at harvest was predicted to superbly control *C. ferrugineus* populations in Oklahoma and Kansas in both 81.6 T and 272.2 MT grain bins over and beyond manual aeration or no aeration at all. There may be an additional benefit from aeration depending on the direction of airflow. For example, Casada and Arthur [64] found that suction aeration was 2–4-times more effective at controlling *C. ferrugineus* and *T. confusum* than pressure aeration, as well as cooling the grain mass [65]. Few studies have documented any trouble related to sanitation issues for either of these methods, though it is likely that poor sanitation will lead to a denser initial assemblage of pests, which may make bulk grain storage susceptible to insect damage if aeration or grain chilling is stopped or if equipment breaks down.

Passive methods of physical control are also relatively robust to poor sanitation regimes because they are usually used in addition to other control tactics (Table 1). Nonetheless, passive methods may still occasionally be susceptible to breakdown. Probably the most important passive physical barrier in stored products is packaging [69]. However, grain inside packaging is susceptible to insect damage, even when treated with an insecticide, and some insects, such as *Prostephanus truncatus* (Horn) (Coleoptera: Bostrichidae) [70], have demonstrated that they can readily chew through packaging [70,71]. Imperfections in packaging as a result of manufacture or as a result of transit in the post-harvest supply chain (reviewed in [72]), can also present additional entry points to insects, especially in areas where pest populations are high and chance of encountering defects is elevated. Scheff et al. [73] found that *P. interpunctella* could invade any package with pinhole punctures, which were able to lay eggs that hatched. Insect populations may even be able to seek out packaged goods because the packaging does not act as an effective barrier to food volatiles used in foraging [74]. Because poorer sanitation will generally result in a greater abundance of insects, the opportunities for a passive physical barrier to become compromised is generally increased.

Active methods may be even more vulnerable to decreased efficacy under poor sanitation regimes than passive methods, because they function similarly to chemical control measures. For example, Arthur [75] found that the presence of flour at the time of application for diatomaceous earth (an inert dust) significantly increased the survival of *T. castaneum* and *T. confusum* by up to a 100-fold. Even amounts of 40–80 mg of flour at the time of application significantly decreased efficacy. That same study found that the availability of food after exposure led to a 3.6–7-fold and 2–23-fold increase in survival of *T. castaneum* and *T. confusum*, respectively, compared to controls without food after a week. Likewise, *T. confusum* exposed to a hydrophobic particle film (kaolinite) and then placed in a dish with 100 mg of flour at low or moderate relative humidity had 1.5–11-fold greater survival at the end of a week compared to control adults given no food [76]. This is problematic because the use of diatomaceous earth and other dusts are one of the few organic control tactics available to food facility managers after harvest. Other active methods such as sieving may be costly, even though they may be more effective at compensating for poor sanitation. Thus, even for active physical methods, enforcing a strict sanitation protocol is valuable for improved efficacy of tactics.

## 5. Sanitation and Biological Control

**Figure 2:**
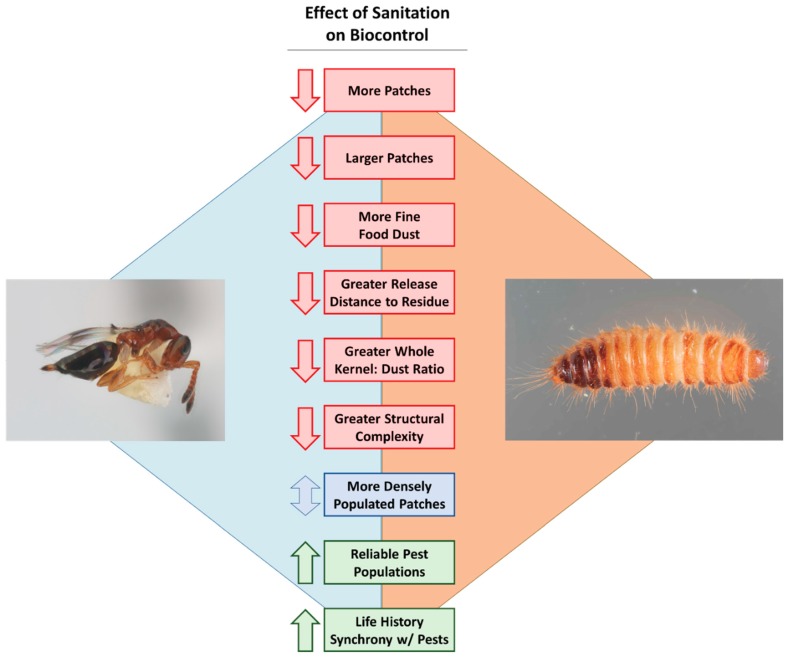
The potential hypothesized negative (red), mixed (blue), and positive (green) effects that sanitation may have on the biological control of post-harvest insects. The parasitoid, *Theocolax elegans* (Hymenoptera: Pteromalidae), and pest, *Trogoderma variabile* are pictured solely for illustrative purposes. Photo credits: Dr. Rob Morrison, USDA-ARS.

While there has been an abundance of work on developing biological control techniques for stored products (e.g., reviewed in [77,78]), relatively little has directly assessed the effect of sanitation on biological control in a rigorous way (113 out of 1647 results). The results that are available indicate that decreased sanitation increases efficacy of biological control by almost 7-fold (Table 1). However, the feedbacks between biological control and sanitation may be more complex (Figure 2), and are still poorly understood. For example, Zdarkova [79] reported that natural enemies were most effective at low pest densities, suggesting that sanitation programs are critical for the successful deployment of natural enemies. The timing of parasitoid releases in controlling pest populations is also undoubtedly important [80]. Parasitoids may be a good alternative to using insecticides on food residues or spillage where concerns about the use of insecticides predominate, usually near the end of the post-harvest supply chain (warehouses and retail stores), and may successfully prevent infestation. One study found that releasing the parasitoid *Habrobracon hebetor* (Say) (Hymenoptera: Braconidae) could prevent the spread of almond moth, *Cadra cautella* (Walker) (Lepidoptera: Pyralidae) from food debris (shelled peanuts, corn, and rolled oats) scattered in a simulated warehouse to adjacent packaged goods [81]. The author found that the parasitoid decreased the percentage of bags infested by 2–7.5-fold and decreased the number of moth larvae per bag by 14–45-fold, depending on type of packaging. Grieshop et al. [82] found that using a combination of *H. hebetor* and *Trichogramma deion* Riley (Hymenoptera: Trichogrammatidae) reduced *P. interpunctella* infestation in cornmeal by almost 6-fold compared to the control, which lacked parasitoids.

While specific research on how biological control is affected by sanitation programs in food facilities has historically been lacking, there are several possible scenarios with different outcomes for how biological control is affected. For instance, if there is an abundance of fine food dust with a larger quantity and size of food patches, one might expect parasitoid foraging efficiency to be decreased (Figure 2). This is because parasitoids may spend more time grooming themselves, have to search more ground to find hosts, and the abundance of food volatiles in the environment may increase their turning frequency and residency time in a given patch (e.g., [83]), even when potential hosts are lacking.

In this situation, a higher density of pests in each patch may make control of pests unrealistic if parasitoids are constrained by the number of eggs they carry, thereby further decreasing the efficacy of biological control. By contrast, an environment with a handful of small patches of low pest density, and less food dust compared to whole seed kernels may result in enhanced efficacy of parasitoids against stored product insects (Figure 2). This may be due to increased searching efficiency and pest encounter rates. These scenarios present testable hypotheses that are worth following up in future research on evaluating the effect of sanitation on biological control efficacy.

## 6. Sanitation and Behaviorally-Based Management

**Figure 3:**
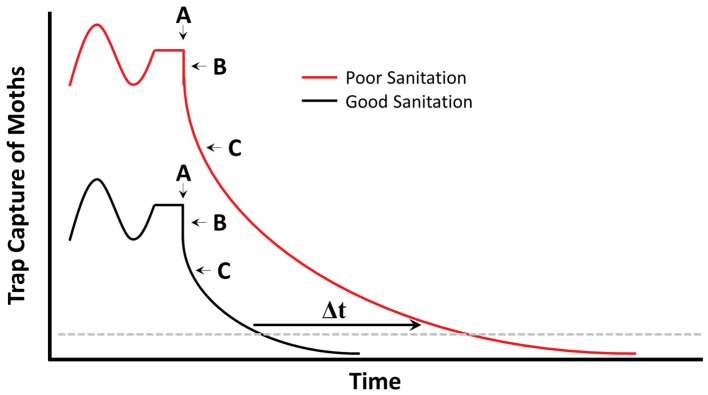
Predicted trap capture of a hypothetical stored product moth population under relatively poorer (red) and better (black) sanitation program. A—implementation of mating disruption program, B—immediate decrease in trap efficiency as a result of stimulus competition (not biologically indicative of decreasing infestation), and C—increasing efficacy of mating disruption over time. The main difference between good and poor sanitation programs is the time (Δt) with which moths decrease below some pre-determined action threshold (dashed grey line).

The only commercially available behaviorally-based management strategy for post-harvest insects currently is mating disruption for *P. interpunctella* and *Ephestia* spp. [84,85,86,87]. While the effect of sanitation on mating disruption has never been specifically examined, our hypothesis is that this tactic may be the most insensitive to decreasing levels of sanitation (Figure 3). Under poor sanitation, one may expect higher initial populations of pests than under an appropriate sanitation regime (e.g., [87]). Immediately after implementation of mating disruption for a pest, we would expect decreased efficiency of pheromone-baited monitoring traps for both scenarios, which is simply an artifact of increased stimulus competition. Facilities with good sanitation programs may expect to lower their pest populations quickly under an action threshold, while those with poorer sanitation would require a longer period to suppress populations (Figure 3). This longer period may result in decreased grain quality, or increased frequency of rejected of commodities, which translates to an increased financial cost. Nonetheless, in the long-term, the tactic should be successful in both cases.

While there are no commercially available mass trapping or attract-and-kill tools, sanitation may also affect the potential future efficacy of such techniques. In other systems, behaviorally-based management strategies such as mass trapping and attract-and-kill are often predicted to break down under high pest pressure [88,89], which in stored products, is more likely to occur under a poor sanitation regime.

## 7. Sanitation and Monitoring Techniques

Substantially less is known about how sanitation affects the efficacy of monitoring with pheromone or kairomone-baited traps. Some research has suggested inconsistent net effects. For example, Nansen et al. [90] evaluated the effect of sanitation on monitoring and spatial distribution for *Sitophilus* spp., *Stegobium paniceum* (L.) (Coleoptera: Ptinidae), and *T. castaneum* in two retail pet stores under areas with high infestation both before and after thoroughly cleaning the areas. Trap captures of *Sitophilus* spp. in one store and *T. castaneum* in the other store increased immediately after sanitation, but equilibrated to levels previously observed prior to sanitation. By contrast, captures of *S. paniceum* in the first store, and *Sitophilus* spp. in the second store, were unaffected by sanitation. In the second store, trap captures of *T. castaneum* and *Sitophilus* spp. were uniformly distributed prior to, but then spatially aggregated after sanitation, whereas *S. paniceum* was randomly distributed on all occasions regardless of sanitation procedures. These were based on single cleaning events, meaning that actual impacts are likely to be cumulative over time for a food facility. Thus, it is unclear what the net effect of sanitation on the distribution of a species may actually be, but in this case, it was ineffective at reducing captures of insects.

However, there is also evidence that sanitation has a strong effect on the efficacy of pheromone- and kairomone-baited monitoring traps. For example, Toews et al. [91] found that simulated warehouses without food patches resulted in almost twice the capture of *T. castaneum* compared to ones with food patches present. Importantly, the correlation between actual density of beetles and traps captures was stronger in warehouses without food patches, highlighting the salience of sanitation programs in food facilities for obtaining reliable information, and potential difficulties in interpreting trap capture when sanitation is ignored.

In fact, Campbell [92] has shown that the landscape pattern of food accumulation on a surface affects the efficacy of traps with and without attractants. Particularly, the study examined how fragmented (small patches) and clumped (large patches) patterns of food accumulation on surfaces in different proportions of total surface coverage affected the efficacy of baited and unbaited traps in capturing *T. castaneum*. Results indicated that while unbaited trap captures were not affected by the pattern of food accumulation on surfaces, pheromone + kairomone baited traps were more effective at capturing adult beetles in fragmented landscapes covering 10% or 30% of the surface area compared to surfaces with no flour or clumped distributions of flour. Romero et al. [93] found that *T. castaneum* was likely to turn more frequently and move more slowly in landscapes with smaller patches of flour than larger ones. Because *T. castaneum* responds relatively weakly to its pheromone and available kairomones, this likely has the effect of keeping the beetle in the vicinity of the trap, increasing the chances that it encounters it and is captured. Similarly, Toews et al. [94] found that presence of food sources adjacent to baited pitfall traps increased capture of beetles in those traps. However, the effects of landscape may vary with different species and different attractants, but has not been rigorously assessed for other post-harvest insects.

**Figure 4:**
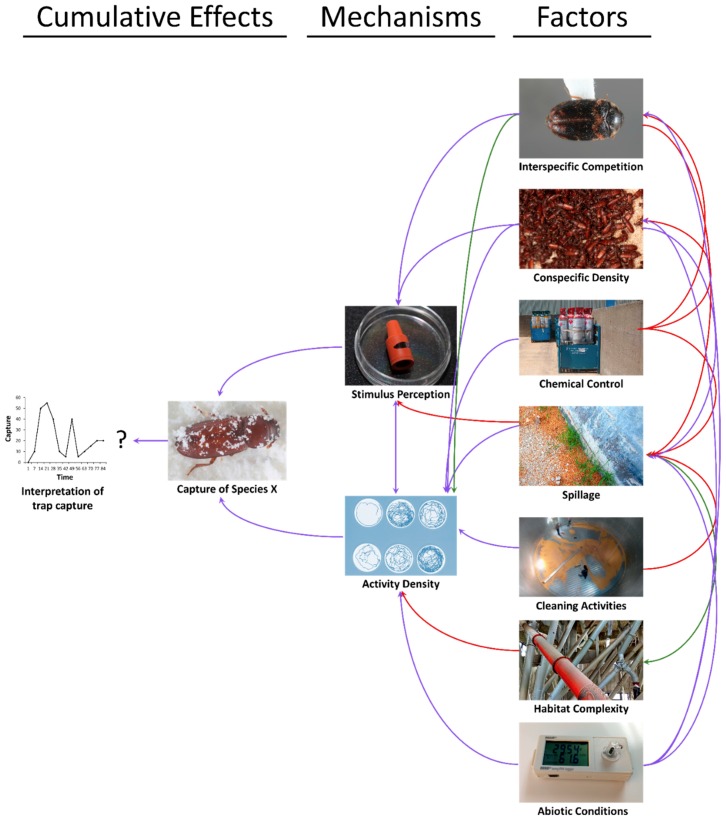
The feedbacks between spillage, environmental variables, and management tactics (factors, right) on pheromone- and kairomone-baited trap captures (cumulative effects, left) through proximate behavioral mechanisms (middle). Factors are hypothesized to have negative (red), mixed (purple), and positive (green) effects on trap capture and its interpretability. Photo credits: Dr. Rob Morrison, USDA-ARS.

Other pest management tactics used in a facility may also influence the interpretability of baited trap captures above and beyond the effects of sanitation alone (Figure 4). Indeed, Toews et al. [95] found that the use of crack and crevice applications in pilot scale warehouses translated to decreased captures in traps. This may suggest to an IPM practitioner that their tactic is succeeding in reducing populations, however direct sampling in that study revealed that populations in beta-cyfluthrin-treated warehouses were increasing at a similar pace to untreated warehouses.

Thus, it is clear that the situation is complex, with multiple feedbacks between the environment, species-specific traits, and management on the level of captures in traps (Figure 4). Changes in pheromone- and kairomone-baited trap captures are behaviorally mediated by changes in movement (velocity, distance moved, turning angle, residency time in an area, etc.) and through perception of trap stimuli, which also feeds back in to affect movement and vice versa (e.g., in the mechanisms section of Figure 4). The magnitude and direction of effects varies over time. For example, the immediate effect of cleaning activities may be to flush out hidden post-harvest insects, resulting in temporary enhancement of captures in nearby traps. However, in the long-term, cleaner areas would be expected to reduce captures in nearby traps. As a result, monitoring traps always need to be evaluated in a context-specific manner in coordination with personnel knowledgeable about activities near traps during the sampling period.

## 8. Sanitation and Other Factors

**Figure 5:**
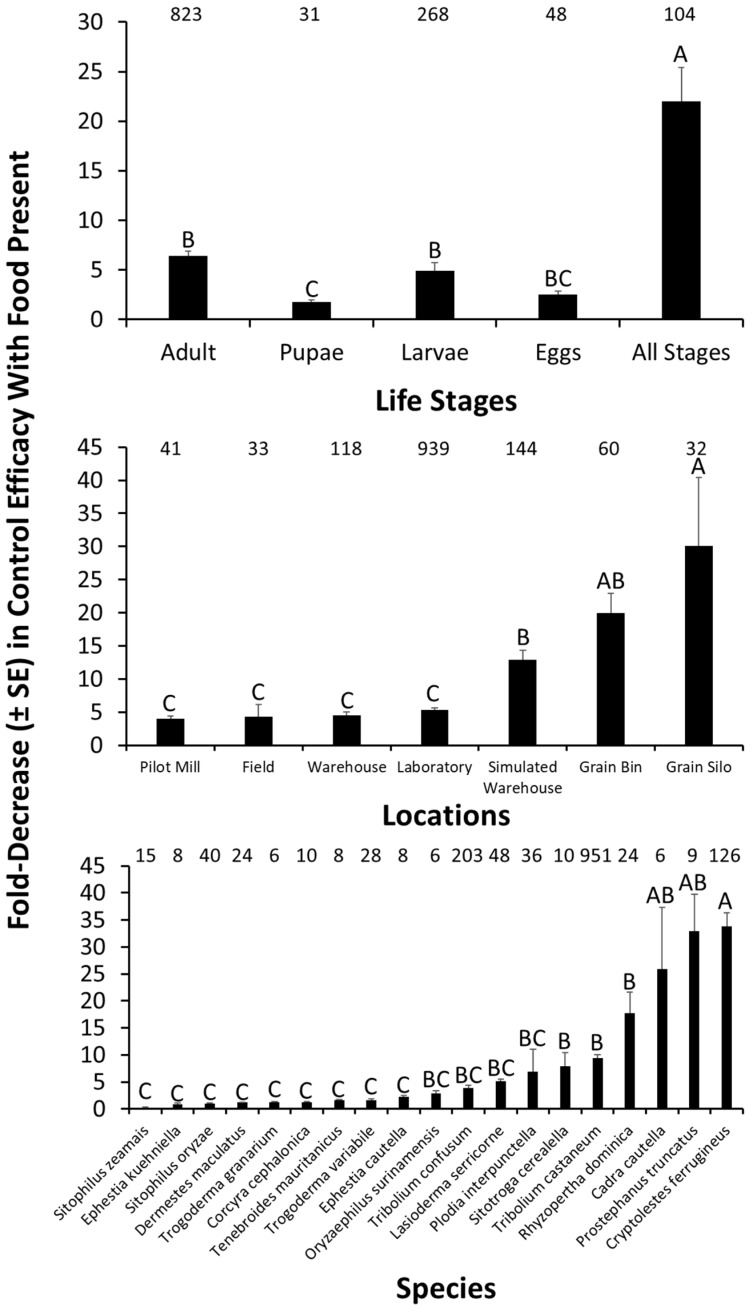
The fold decrease in control efficacy in the presence of food compared to controls with no or less food for different life stages (top panel) of stored product insects, assessed in different locations (middle panel), for a variety of target species (bottom panel). Numbers located above bars represent the number of independent observations from experiments in the literature. Bars with shared letters are not significantly different from each other (Tukey HSD, α = 0.05). Factors with fewer than *n* = 3 observations were excluded from graphs and analysis.

In addition to the control measures discussed above, there are a variety of other factors that may intersect with IPM of stored product insects, and modulated the effect of sanitation on control efficacy. For example, there may be significant life stage-specific decreases in efficacy of control under poorer sanitation regimes compared to better ones (*F* = 26.0; df = 4, 1170; *p* < 0.0001; Figure 5). The most susceptible life stage to changes in sanitation were adults, which experienced a 6.4-fold decrease in tactic efficacy in the presence of food compared to its absence. The least susceptible life stage to changes in sanitation were pupae. When mixed stages were considered, populations of stored products insects as a whole were 3-times more susceptible to decreases in the efficacy of control measures under poorer sanitation regimes compared to the most susceptible life stage (e.g., adults). Across tactics, it is clear that stored product insects are susceptible in a life-stage-dependent manner to changes in sanitation regime.

Likewise, the effects of sanitation are significantly modulated by location of management (*F* = 5.08; df = 6, 1170; *p* < 0.001; Figure 5). Regardless of location, control efficacy decreased by 4–30-fold under poorer sanitation. The greatest decrease in efficacy under poorer sanitation regimes occurred in simulated warehouses, grain bins, and grain silos, where sanitation was 3-, 5-, and 7-times more impactful on control tactic efficacy than in pilot-scale mills. Some structures may be more or less structurally complex, which may add to the variation in how effective sanitation measures may be. Food facilities with more structural refugia may already be at a disadvantage in allowing insect populations to more easily build up, and thus the effect of sanitation may be more pronounced in a simpler habitat such as a grain bin or silo. However, regardless of where a management tactic is implemented, sanitation is likely to have a dramatic effect on its efficacy.

Finally, sanitation conditions significantly affected the efficacy of controlling target stored product species to varying degrees (*F* = 21.4; df = 18, 1170; *p* < 0.0001; Figure 5). The top five stored product insects most affected by sanitation conditions are *C. ferrugineus*, *P. truncatus*, *C. cautella*, and *R. dominica*, whose control decreased by 9–34-fold under poorer sanitation conditions. However, control efficacy decreased by at least 2-fold for 72% of the species for which data are available. In some cases, there are a low number of observations, making firm conclusions about any of the specific species tentative, except for those where there are ample independent observations. Nonetheless, stored product species are broadly protected from control tactics under poor sanitation.

## 9. Gaps in Knowledge and Conclusions

It is clear from the data presented in this review that sanitation is incredibly important for the efficacy of a large variety of IPM tactics in managing stored product insects. We found that sanitation had the largest deleterious effect on tactics that could not or had difficulty penetrating the surface of grain. In these cases, grain may act as a refuge, and may allow insects to help cope with the management action. Even for tactics where sanitation does not directly impact immediate efficacy or even improves it, there may be complicating considerations that reduce the usefulness of the tactic, for example with grain protectants and packaging. Poor sanitation in these cases will result in refugia in grain bins, which provides the opportunity for pests to re-infest the commodity, or else improves the chances that insects encounter defects in packaging. Poor sanitation may indirectly decrease the efficacy of biological control programs, and even affect the interpretability of pheromone- or kairomone-baited trap captures, thereby complicating decision-making at a food facility. At every step in the pest management process, it is clear that poor sanitation has cascading negative effects.

These deleterious outcomes can be easily avoided if proper sanitation programs are implemented and consistently followed. Common best practices include: removing sites of spillage or food dust accumulation (sweeping, vacuuming) regularly, cleaning bins and silos after grain is off-loaded, monitoring the surrounding environment for spillages, especially around rail lines associated with a storage facility, physically separating the commodity from the environment and ensuring it is well-sealed, rotating inventory in retail stores, checking for pest infestations on incoming commodities for exclusion, and ensuring regular emptying and cleaning inside and outside trash receptacles associated with the facility. In addition to removing spillage, disposal of residue is necessary so that insects do not return to the facility. A diversified IPM program that includes a strong sanitation component as a foundation is likely to be more resilient to potential insect infestations. Future work needs to address the specific effects of sanitation and associated processes on the efficacy of 1) biological control, 2) behaviorally-based management strategies, and 3) monitoring programs based on some of the hypotheses generated in this review. It is likely that further work will serve to highlight the continuing importance of sanitation for every aspect of stored product protection.

## Figures and Tables

**Table 1:** Summary of net effect of decreased sanitation (presence of food or presence of more food compared to none or less) on the efficacy of a variety of IPM tactics to management stored product insects in food facilities. Effects are denoted as negative (−), positive (+), neutral (0), or mixed (−/+).

Type of Control	Tactic	Decreased Sanitation Impact on Efficacy					
		Direct Effects					Indirect Effects
		Effect Direction	*n*	Avg. Fold-Δ ^1^	±	SE	Effect Direction ^2^
Chemical Control		−	1240	8.5	±	0.39	−
	Fumigation	−	67	15.7	±	2.04	−
	Grain Protectants	−	100	1.3	±	0.18	−
	Aerosols	−	389	2.8	±	0.19	−
	Residual Treatments	−	656	10.5	±	0.56	−
	Modified Atmospheres	−	28	1.1	±	0.11	−
Physical/Cultural		−	294	17.1	±	1.76	−
	Heat Treatment	−	73	13.6	±	4.35	−
	Grain Chilling/Aeration	+	132	31.5	±	2.48	−
	Passive Methods (packaging)	−	64	1.6	±	0.07	−
	Active Methods (DE, inert dusts)	−	25	3.5	±	1.33	−
Biological Control		+	113	6.8	±	1.8	−/+
Behaviorally-Based		0/−		na ^3^		na	0/−
	*Mating Disruption*	0		na		na	−
Stimuli-Based Monitoring		−/+		na		na	−/+

^1^ Average fold-change in efficacy of the tactic in the presence of food relative to less or no food. ^2^ Hypothesized indirect effects based on prior literature. ^3^ Not applicable because no data are available; direction of direct effects and indirect effects is hypothesized.

## Supplementary Materials

The following are available online at https://www.mdpi.com/2075-4450/10/3/77/s1, Table S1: Full compendium of citations consulted in constructing MS, including for quantitative analysis.
